# Flecainide-Induced Acute Respiratory Distress Syndrome: A Case Report

**DOI:** 10.7759/cureus.61637

**Published:** 2024-06-04

**Authors:** Noah R Schneegurt, Christian Wright, Neil Glenn, Inderpal Thethi

**Affiliations:** 1 Internal Medicine, Mount Carmel Health System, Grove City, USA; 2 Pulmonary and Critical Care Medicine, Mount Carmel Health System, Columbus, USA

**Keywords:** diffuse lung injury, drug-induced lung injury, flecainide-induced lung injury, ards, flecainide

## Abstract

Flecainide is an antiarrhythmic drug that rarely causes lung injury. We present a case of flecainide-induced lung injury (FILI) that resulted in acute respiratory distress syndrome (ARDS) and resolved after flecainide discontinuation and corticosteroid treatment. FILI has been shown to occur days to two years after treatment initiation. Our presented case shows that FILI can occur after at least five years of therapy and is the first to show lung injury after a period of flecainide cessation and subsequent re-initiation. Clinical impacts may be large, as flecainide becomes more commonplace in medical pharmacopeia.

## Introduction

Flecainide-induced lung injury (FILI) is a known, but rare, clinical phenomenon that can present with nonspecific pneumonitis and acute respiratory distress syndrome (ARDS). We present a case of flecainide-induced ARDS that required intensive care monitoring in a community hospital setting. The patient improved after flecainide cessation and corticosteroid administration.

Flecainide is a class Ic antiarrhythmic medication used for atrial and ventricular arrhythmias, most notably atrial fibrillation or flutter. The mechanism of action is cardiac cell membrane sodium channel inhibition that results in a shorter action potential duration and prolongation of atrial refractory periods, which can prevent atrial fibrillation [1]. Important side effects include atrial flutter with 1:1 atrioventricular conduction, ventricular arrhythmias, and worsening of congestive heart failure with reduced ejection fraction [2]. Flecainide has seen a tripling in yearly prescriptions since 2013 [3].

Drug-induced lung injury is a well-known side effect of numerous medications, most commonly cytotoxic chemotherapy agents, methotrexate, targeted biologic agents, nitrofurantoin, and amiodarone. It is typically suspected when there is a temporal relationship between drug exposure and the onset of symptoms, the absence of a more likely cause, and improvement with the withdrawal of the medication. There is no unifying histopathological appearance or mechanism of injury between the various groups of medications. Lung injury is often reversible with discontinuation of the causative agent and/or glucocorticoid therapy, although many patients do not recover and develop pulmonary fibrosis or enter florid respiratory failure [4].

## Case presentation

**Figure 1:**
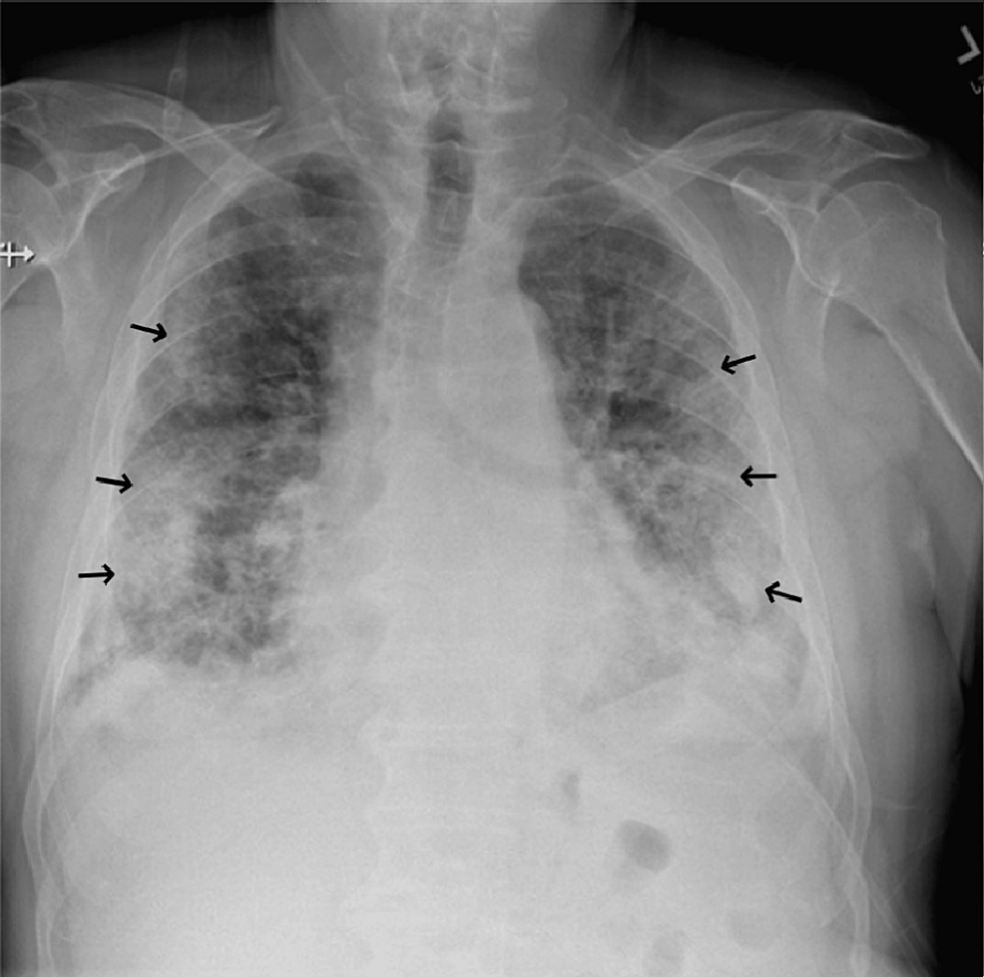
Posterior-anterior chest X-ray on arrival showing bilateral parenchymal infiltrates

**Figure 2:**
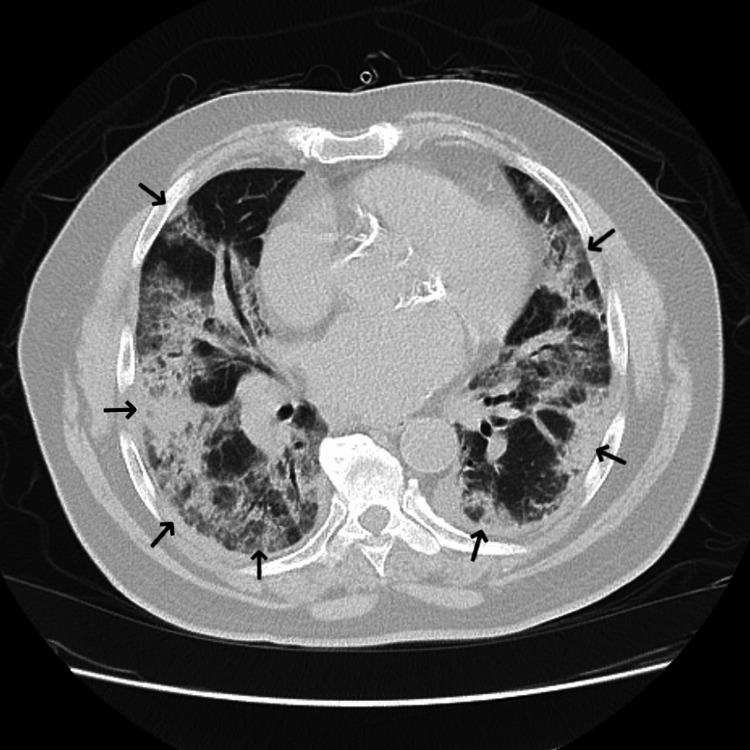
An axial slice of the computed tomography pulmonary angiogram on day 1 of admission showing diffuse bilateral infiltrates

A 74-year-old male with a history of paroxysmal atrial fibrillation treated with atenolol, diltiazem, and flecainide presented to the emergency department for evaluation of progressively worsening dyspnea and dry cough for approximately two weeks. He had been prescribed twice-daily immediate-release flecainide acetate 100 mg (2.1 mg/kg/day) for at least five years prior to presentation. He was also using an albuterol inhaler, cholecalciferol supplementation, lisinopril, pantoprazole, rivaroxaban, and sertraline prior to admission. Physical examination revealed decreased breath sounds in the right lung base, regular heart rate and rhythm, and no peripheral edema. Laboratory evaluation was notable for a leukocytosis of 16.0 K/mcL consisting of 87.1% neutrophils and 4.7% lymphocytes with a relative proportion of monocytes, eosinophils, and basophils within normal limits. An indeterminate B-type natriuretic peptide with a cardiac troponin level within normal limits, both markers of cardiac dysfunction, suggested a noncardiac cause of his dyspnea. The elevated B-type natriuretic peptide level suggests congestive heart failure: an indeterminate B-type natriuretic peptide level is one in which congestive heart failure is neither suggested nor excluded. A posterior-anterior chest X-ray on presentation revealed diffuse parenchymal and interstitial infiltrates bilaterally (Figure 1). Computed tomography pulmonary angiogram (CTPA) showed diffuse infiltrates bilaterally with no acute pulmonary embolism (Figure 2). CTPA was performed to rule out acute pulmonary embolism given the patient's hypoxemia on presentation. No further chest computed tomography was performed due to the characteristic chest X-ray findings of acute respiratory distress syndrome. The electrocardiogram confirmed normal sinus rhythm.

The patient was admitted and started on empiric antibiotics. A transthoracic echocardiogram demonstrated preserved left ventricular systolic function and normal ventricular wall thickness. Comprehensive infectious workup was negative, including *Streptococcus pneumoniae *and *Legionella pneumophila *urinary antigens, respiratory virus panel molecular study, aspergillus antigen, bronchoalveolar lavage bacterial, fungal, and acid-fast bacilli cultures, bronchoalveolar lavage gram stain, urine culture, and multiple sets of blood cultures. Right middle lobe bronchoalveolar lavage revealed pink fluid with 8,552 cells/mL red blood cells and 71 cells/mL total nucleated cells, with 68% being neutrophils, 13% lymphocytes, 18% monocytes, and 1.0% eosinophils. There was no *Pneumocystis jirovecii* or malignant-appearing cells in the bronchoalveolar wash.

After several days, the patient required intubation for worsening hypoxemia and started on stress-dose steroids. Repeat chest X-ray was consistent with ARDS. The arterial oxygen partial pressure to fractional inspired oxygen ratio (P/F ratio) was 194 mmHg, suggesting moderate ARDS. The patient never required prone positioning or paralysis. Antinuclear and antihistone antibodies both resulted in positive. C-reactive protein and erythrocyte sedimentation rates were elevated to 27.2 mg/dL and 17 mm/hr, respectively. A diagnosis of flecainide-induced lung injury was made as a diagnosis of exclusion and flecainide was discontinued. The diagnosis of flecainide-induced lung injury was supported by the patient's improvement after drug discontinuation. Glucocorticoid therapy was also initiated with intravenous methylprednisolone, 60 mg every 6 hours (2.5 mg/kg/day), which was increased to 125 mg every 6 hours (5.2 mg/kg/day) after 2 days of treatment. The methylprednisolone was then tapered over 18 days.

The patient went into atrial fibrillation while intubated. His pulmonary status gradually improved, and he was ultimately extubated. Cardioversion was later performed and sotalol was initiated with the maintenance of sinus rhythm. He was not discharged with glucocorticoids.

## Discussion

Rare cases of FILI have been reported as early as 1991 [5]. Presentation is typically new-onset respiratory distress and may progress to intubation and death. The time course is variable, with lung injury reported anywhere from hours [5] to months [6] after initiation. Our patient had the onset of ARDS after a minimum of five years of flecainide therapy, years longer than what Godil et al. presented in 2020 [7].

The FILI etiology is unknown. Cell-mediated damage has been suggested [8] and supported by documented bronchoalveolar lavages and lung biopsies demonstrating lymphocytic and eosinophilic inflammation [6]. Bronchoalveolar lavage reported by Moureau did not show lymphocytic predominance. Lung biopsy has previously demonstrated cryptogenic organizing pneumonia [8,9]. Flecainide accumulation in lung tissues for an extended time after cessation is established knowledge from autopsy studies, though the impact remains unclear [8,10]. There are no reports on serum levels of flecainide in regard to the incidence of drug-induced lung injury, although Moureau reported a plasma flecainide concentration of 32.2 μg/L with a corresponding lung tissue flecainide concentration of 1.6 μg/g. Plasma flecainide concentration is not frequently performed in clinical settings.

Flecainide discontinuation and corticosteroids are the most common therapies with good responses in most cases. Glucocorticoids are typically prescribed and have been continued for as many as three months, although the clinical impact of glucocorticoids is unclear. Improvement in radiographic appearance in surviving patients has been reported throughout the literature [5,6].

## Conclusions

We presented a case of FILI with a subacute to acute presentation resulting in ARDS and mechanical ventilation that resolved after flecainide cessation and corticosteroids. Flecainide is a known, but rare, cause of acute and subacute lung injuries that can arise after hours and up to at least five years after therapy initiation. Treatment consists of flecainide discontinuation and, typically, glucocorticoids with good response and eventual resolution of most radiographic evidence of disease. As more cases of FILI are reported and flecainide sees increasing prescriptions, it will be important to investigate risk factors and provoking events that initiate an episode of FILI after months to years of stable therapy.
